# Sustainable Water-Resistant Cotton Fabrics Coated with a Chitosan/Wax Composite Extracted from Discarded Outer Cabbage Leaves

**DOI:** 10.3390/molecules31101611

**Published:** 2026-05-11

**Authors:** Walaikorn Nitayaphat, Kageeporn Wongpreedee, Thanut Jintakosol

**Affiliations:** 1 Department of Chemistry, Faculty of Science, Srinakharinwirot University, Bangkok 10110, Thailand; 2 College of Creative Industry, Srinakharinwirot University, Bangkok 10110, Thailand; kageeporn@g.swu.ac.th

**Keywords:** plant-based wax, cabbage leaf waste, chitosan, cotton fabric, water-resistant coating, bio-based coating

## Abstract

The water-resistant properties of a chitosan/OCL wax composite were evaluated on cotton fabrics using a dip-coating method. The plant-based wax was extracted from discarded outer cabbage leaves. The influence of the chitosan-to-OCL wax weight ratio on textile properties—including wettability, air permeability, mechanical performance, and stiffness—was systematically investigated. In addition, laundering tests were conducted to assess the durability of the coating. The results demonstrated that the cotton fabric coated with a chitosan/OCL wax composite at a 70/30 weight ratio exhibited the highest hydrophobicity, achieving a water contact angle of 157.87°. The coated cotton fabrics also showed good washing stability. Measurements of bending length and flexural rigidity revealed that cotton fabrics coated with the chitosan/OCL wax composite exhibited greater stiffness than the untreated samples. The combined use of chitosan and OCL wax provided a synergistic enhancement in water-resistant performance. These findings highlight the potential of the chitosan/OCL wax composite as a non-toxic and environmentally friendly finishing agent for cotton fabrics.

## 1. Introduction

The management of large volumes of food waste remains a significant global challenge. Within the framework of a circular economy, efforts are directed toward minimizing waste and promoting the continuous use of resources by converting waste streams into renewable feedstocks for value-added products. From both sustainability and environmental perspectives, the utilization of waste derived from renewable resources is preferred over the depletion of finite materials. Consequently, research on biodegradable polymer composites has attracted increasing attention in recent years [1,2]. In this context, food and agricultural waste represent promising and sustainable raw materials for the development of bio-based products. Various studies have explored their application in biocomposites for construction (e.g., citrus limetta peel, orange peels, and eggshells) [3,4,5], food packaging (e.g., almond shells, grape stalk) [6,7], agricultural inputs (e.g., cassava bagasse) [8], tissue engineering and wound healing (e.g., chitosan) [9], and furniture (e.g., flax shive) [10]. However, to the best of the authors’ knowledge, limited research has been conducted on the use of agricultural waste-based biocomposites for imparting water-resistant properties to textiles.

Cabbage (*Brassica oleracea* L. var. *capitata* L.) is one of the important leaf vegetables with significant economic value and can be cultivated year-round. In Northern and Northeastern Thailand, its popularity has increased, allowing 3–4 harvests annually [11]. It is rich in various bioactive compounds, including vitamin C, glucosinolates, carotenoids, flavonoids, and polyphenols [12,13,14,15]. The edible part consists mainly of the leaves, divided into cabbage heads and outer cabbage leaves (OCL). Commercially, only the cabbage heads are consumed, while the OCL and roots are discarded during production, and distribution, representing about 30% of the total yield and contributing to substantial waste [16]. Efforts to valorize this biomass have focused on producing biochar [17,18], cosmetic ingredients [19], functional food additives [20,21,22], and nanocellulose [23] from the discharged outer leaves, turning waste into valuable resources. The OCL are typically green to dark green due to a surface wax layer that regulates non-stomatal water loss [24] and provides hydrophobic protection against dust, bacteria, and fungi [25]. This wax consists mainly of esters of very long-chain fatty acids and alcohols, along with alkanes, aldehydes, ketones, and both primary and secondary alcohols [26,27]. Given its composition, cabbage leaf wax is suitable for extraction and utilization. Wax extracted from discarded OCL offers a sustainable, high-value application for this underused by-product.

Cotton fabric is widely used in daily life due to its exceptional properties, including breathability, softness, comfort, flexibility, mechanical strength, renewability, affordability, warmth, and biodegradability. However, the abundance of hydroxyl groups on the surface of cotton fibers makes them naturally hydrophilic, which restricts their use in applications involving water exposure [28,29]. To overcome this limitation, surface modification is necessary to introduce hydrophobic characteristics to cotton, making the development of effective hydrophobization methods essential. Various approaches have been employed to impart hydrophobicity to cotton and expand its functional range.

Fluorocarbon-based water repellents exhibit excellent hydrophobic performance; however, they pose significant environmental concerns due to their persistence, bioaccumulative nature, and adverse ecological impacts [30,31]. Consequently, the development of fluorine-free and environmentally friendly water-repellent agents has become increasingly important from both environmental and safety perspectives [32,33,34]. Paraffin-based water repellents are economically favorable, but their relatively poor performance often necessitates blending with polyacrylates or melamine resins to achieve satisfactory results [35]. Polyacrylate-based agents are widely used because of their good washing durability and cost-effective processing; however, they may cause fabric hand-hardening and are susceptible to surface scratching [36]. Polyurethane-based agents offer the most balanced overall performance, although their practical use is limited by their relatively high cost [37]. Silicon-based treatments are often considered a more environmentally friendly alternative, particularly the microgel preparation process, which has gained significant attention in the cotton textile industry for achieving water-repellent properties [38]. However, some silicon compounds can still be toxic and harmful to the environment [39,40]. Moreover, the covalent binding or adsorption of inorganic materials can reduce the biodegradability of cellulose, thereby contributing to textile waste. Silicon-based coatings also tend to exhibit limited durability, and the treatment processes often involve harsh and toxic solvents, raising additional environmental and safety concerns.

This study aimed to develop a biodegradable, fluorine-free, and waterproof contact material utilizing wax extracted from discarded OCL. Different concentrations of the extracted wax were dispersed in chitosan solutions to prepare stable suspensions. Chitosan, a nontoxic, biocompatible, and biodegradable polymer, served as an emulsifier, stabilizing the wax through electrostatic interactions between the positively charged chitosan and the negatively charged wax [41,42,43]. The resulting suspensions were applied to cotton fabric, and the coated samples were analyzed for color characteristics, surface morphology, wettability, and mechanical properties. Overall, this work demonstrates the potential of combining chitosan and discarded OCL-extracted wax for developing environmentally friendly, water-resistant fabrics.

## 2. Results and Discussion

### 2.1. Chemical Structure

**Figure 1:**
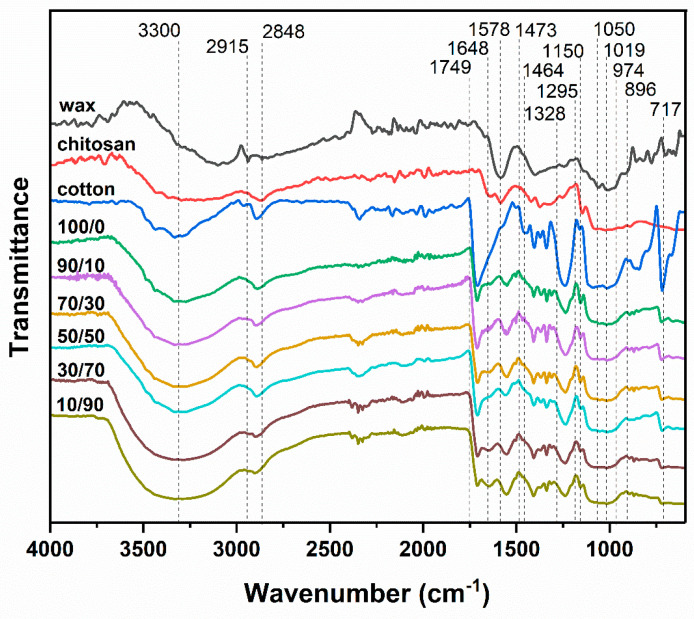
FTIR spectra of chitosan, OCL wax, uncoated cotton fabric, and cotton fabrics coated with a chitosan/OCL wax composite.

The FTIR spectra of chitosan, OCL wax, uncoated cotton fabric, and cotton fabrics coated with a chitosan/OCL wax composite are presented in Figure 1.

The FTIR spectrum of OCL wax exhibited prominent absorption bands at 2915 and 2848 cm^−1^, assigned to the asymmetric and symmetric stretching vibrations of C-H bonds in long aliphatic hydrocarbon chains [44,45], which matches well with cellulose C-H and CH_2_ signals [46], but in terms of visual appearance are significantly different with the pronounced shape of the two peaks. Unique to wax is the signal at 1749 cm^−1^, which corresponds to the carbonyl group of the ester linkage in fatty acids [44,45]. Like peaks observed around 2900 cm^−1^, the two peaks at approximately 1470 cm^−1^ are characteristic of wax, although they are also present in cellulose. These absorptions are attributed to C-H (CH_2_) bending, scissoring, and rocking vibrations [44,45]. The region between 1150 and 1019 cm^−1^ is commonly referred to as the fingerprint region, mainly corresponding to various C-O linkages [46]. The absorption bands observed at 720 cm^−1^ are attributed to vibrations associated with non-planar amide groups.

For the chitosan, the broad absorption band in the 3000–3500 cm^−1^ region is associated with the stretching vibrations of free hydroxyl (-OH) groups as well as the symmetric and asymmetric stretching vibrations of N-H bonds in the amino groups of chitosan molecules [47]. The doublet observed at approximately 2880 cm^−1^ is attributed to the asymmetric and symmetric stretching vibrations of C-H bonds in chitosan [48]. The presence of N-acetylglucosamine units is evidenced by characteristic absorption bands corresponding to C=O stretching (amide I) at 1648 cm^−1^, N-H bending (amide II) at 1578 cm^−1^, and N-H combined bending and stretching vibrations (amide III) at 1295 cm^−1^ [49]. In addition, the absorption bands at 1170 and 1023 cm^−1^ are assigned to symmetric C-O-C stretching vibrations associated with the pyranose ring structure of chitosan [50].

The FTIR spectrum of the uncoated cotton fabric exhibited characteristic absorption bands of cellulose. A broad band centered around 3300 cm^−1^ is attributed to –OH stretching vibrations, representing the most prominent feature of cellulose. The absorption band at 2898 cm^−1^ corresponds to C-H stretching vibrations within the cellulose backbone [51]. The CH_2_ bending vibration was observed at 1430 cm^−1^. The band at 974 cm^−1^ is assigned to C-O stretching vibrations of xylan, while the absorption at 896 cm^−1^ is associated with the β-glycosidic linkages of cellulose [52]. Additionally, the band observed near 717 cm^−1^ is attributed to CH_2_ rocking vibrations [44,45,46].

After coating with the chitosan/OCL wax composite, characteristic FTIR absorption bands attributable to both components were observed. The intensities of these bands varied according to the chitosan/wax ratio on the cotton fabric surfaces. In particular, the wax-related bands at 2915, 2848, 1749, 1470, and 720 cm^−1^ were notably intensified in the 90/10, 30/70, 50/50, 70/30, and 10/90 samples, confirming the successful deposition of wax onto the cotton fabric surfaces.

### 2.2. Appearance and Colorimetric Data

**Figure 2:**
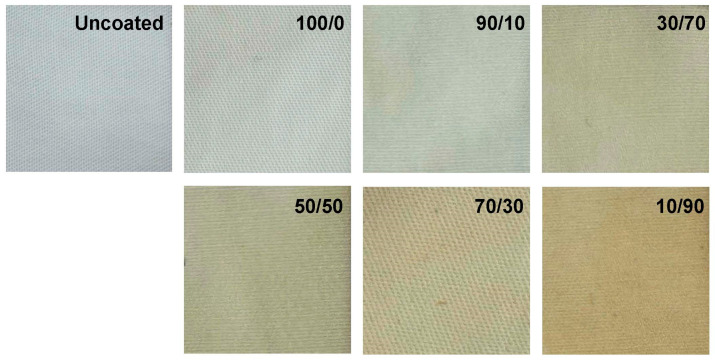
Visual appearance of cotton fabrics coated with a chitosan/OCL wax composite.

A photograph of the uncoated and coated cotton fabrics is shown in Figure 2, while Table 1 summarizes the color parameters ($L^{*}$, $a^{*}$, $b^{*}$), whiteness index (WI), yellowness index (YI), and the color difference (∆E) between the uncoated and chitosan/OCL wax composite coated samples.

The uncoated cotton fabric exhibits a distinctly white appearance. After coating with chitosan (100/0), the lightness ($L^{*}$) decreased, accompanied by increases in both $a^{*}$ (redness) and $b^{*}$ (yellowness) values. These changes are attributed to the intrinsic color of chitosan. Accordingly, the 100/0 sample showed a lower WI and higher YI than the uncoated fabric. This behavior likely results from pore filling by chitosan and possible reactions of its functional groups, which promote yellowing [53,54,55]. Coating with the chitosan/OCL wax composite produced an even more yellowish tone. Increasing wax loading progressively decreased WI and increased YI, as reflected by higher $b^{*}$ values, indicating a concentration-dependent effect. Overall, the application of chitosan and OCL wax altered the fabric color, which is further confirmed by the (∆E) values between the coated and uncoated samples.

### 2.3. Morphological Study

**Figure 3:**
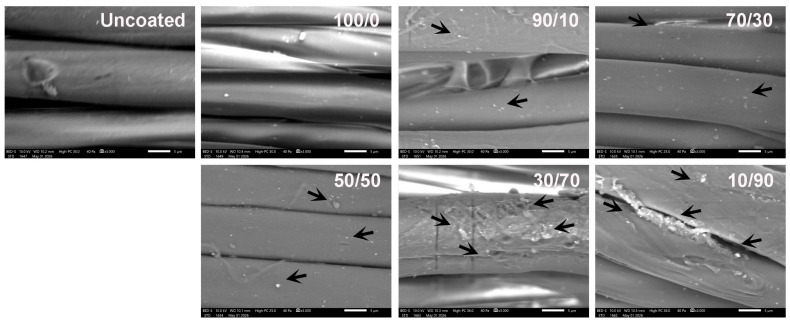
SEM photographs of cotton fabrics coated with a chitosan/OCL wax composite. An arrow is used to indicate the location of the wax of the fabric surface.

Scanning electron microscopy (SEM) is commonly employed to visually examine the surface morphology of solid materials [56]. This technique generates high-resolution images by scanning the sample surface with a focused beam of electrons. In this study, SEM was used to investigate the surface morphology of uncoated and coated cotton fabrics in order to evaluate the effect of chitosan/OCL wax composite treatment on fiber structure. The corresponding micrographs are presented in Figure 3.

The characteristic natural convolutions of uncoated and coated cotton fibers are evident in the SEM images. The uncoated cotton fabric exhibits a relatively smooth surface, which may result from the removal of non-cellulosic components, such as hemicellulose, lignin, waxes, and pectin. Cavities between the cellulose fibers are also observed. In contrast, the chitosan coated cotton fabric (100/0 sample) shows a slightly rougher surface compared to the uncoated sample, owing to the deposition of chitosan and the formation of an adhered film covering the fiber surface. This effect can be explained by electrostatic interactions between the negatively charged cellulose fibers and the positively charged chitosan molecules. Such behavior is consistent with previous reports indicating that chitosan initially penetrates and fills surface pores, followed by the formation of a thin coating layer [57]. After the application of chitosan/OCL wax composite coatings, wax particles were observed on all fabric surfaces, and their number increased with increasing wax concentration. In the 50/50 sample, cavities were detected within the fabric structure, likely due to the insufficient chitosan content to effectively occupy and bind the fabric interstices. Furthermore, coatings with higher wax content exhibited noticeable wax aggregation, which may result from the limited amount of chitosan available to adequately disperse and stabilize the wax phase. Moreover, the fabrics were dried at 100 °C after coating, a temperature exceeding the melting point of natural wax (about 65–84 °C) [58,59,60]. This drying condition may have caused the wax to melt, promoting its redistribution and aggregation on the fabric surface, thereby contributing to the formation of wax agglomerations.

### 2.4. Air and Water Vapor Permeability

**Figure 4:**
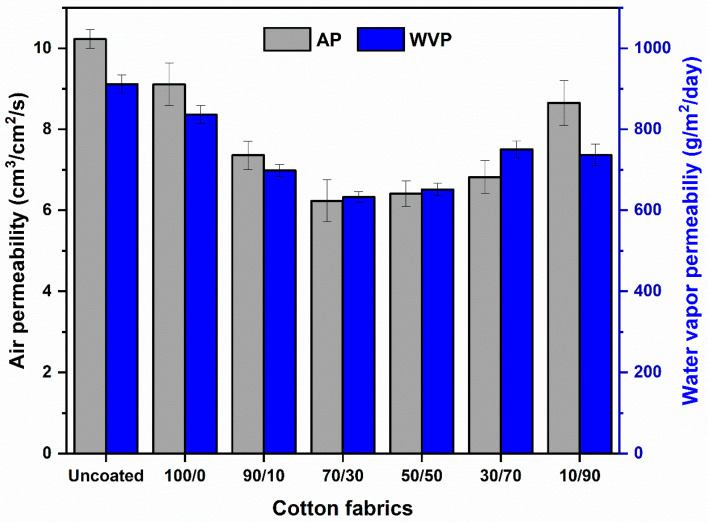
Air permeability and water vapor permeability of cotton fabrics coated with a chitosan/OCL wax composite.

The permeability of air and moisture definitely affects the comfort of human body [61]. Fabrics with high air and moisture permeability enhance clothing comfort by maintaining a cool, dry, and thermally balanced skin microclimate. The air permeability and water vapor permeability of uncoated cotton fabric and cotton fabrics coated with a chitosan/OCL wax composite are presented in Figure 4.

As presented in Figure 4, the uncoated cotton fabric showed the highest air permeability and water vapor permeability (WVP), with values of 10.23 cm^3^ cm^−2^ s^−1^ and 911.63 g m^−2^ day^−1^, respectively. This performance reflects the inherent breathability and moisture-management capability of cotton, making it well suited for garment applications. After chitosan coating, both air permeability and WVP decreased to 9.11 cm^3^ cm^−2^ s^−1^ and 836.49 g m^−2^ day^−1^, respectively. This reduction indicates that the chitosan layer significantly influenced the transport properties of the cotton fabric. The decrease can be attributed to the formation of a crosslinked thin-film layer on the fabric surface, which partially blocks the pore structure and acts as a barrier to air and moisture transmission. A further decline in permeability was observed with increasing wax content in the coating. This behavior is attributed to greater surface coverage by the wax phase, which increases the continuity and density of the crosslinked film and further restricts air and vapor diffusion through the fabric structure [62,63]. Moreover, the high WVP of the uncoated fabric is associated with the hydrophilic nature of cotton, whereas the coated fabrics exhibited reduced hydrophilicity, as shown in Figure 4. Among the coated samples, the 70/30 composition displayed the lowest air permeability and WVP (10.23 cm^3^ cm^−2^ s^−1^ and 911.63 g m^−2^ day^−1^, respectively) and the highest water contact angle, indicating the greatest hydrophobicity.

### 2.5. Water Contact Angle

The water contact angle (WCA) value is an important parameter used to evaluate the hydrophilicity or hydrophobicity of fabrics, providing information about the surface properties and wettability of the fabric. A hydrophobic surface, characterized by a WCA greater than 90°, tends to repel water and reduce water absorption. In contrast, a WCA lower than 90° indicates a hydrophilic surface with higher wettability [64,65,66]. Typically, degree of WRA depends on the length of aliphatic chain—specifically, an increase in the number of carbon atoms enhances hydrophobicity [67]. In addition, the presence of a crosslinked network structure contributes to improved water resistance, leading to higher WCA values [68]. Generally, the WCA is affected by the length of the aliphatic chain, where an increase in the number of carbon atoms leads to enhanced hydrophobicity [66]. Additionally, the formation of a crosslinked network structure contributes to improved water resistance, thereby increasing the contact angle [69].

**Figure 5:**
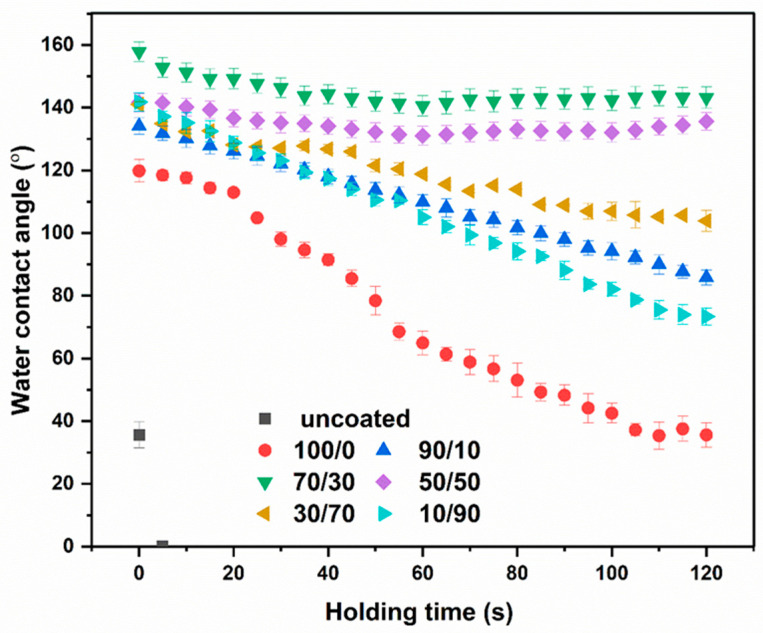
Water contact angle of cotton fabrics coated with a chitosan/OCL wax composite.

**Figure 6:**
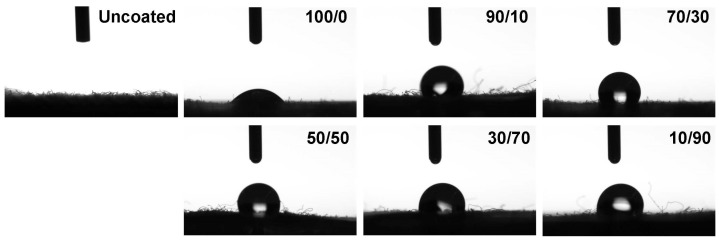
Microscopic images of water droplets on the surface of cotton fabrics coated with a chitosan/OCL wax composite, measured at a holding time of 60 s.

The water resistance of the fabric surface was assessed using WCA measurements (Figure 5) and microscopic images of water droplets (Figure 6). The uncoated cotton fabric exhibited a contact angle of 35.65°, indicating a hydrophilic surface that readily allows water to spread. This behavior is attributed to the cellulose structure, which contains abundant hydroxyl groups capable of forming hydrogen bonds with water molecules. Consequently, cotton fabrics are easily and rapidly wetted by water and other liquids, making them more susceptible to staining [70,71]. After coating with chitosan, the WCA increased to 119.82°, indicating that the chitosan layer rendered the fabric surface hydrophobic. This effect can be attributed to the increased surface roughness introduced by the chitosan coating on the cotton fibers. In addition, during film solidification, molecular rearrangement of chitosan occurs, in which functional groups such as hydroxyl and amine groups tend to align parallel to the film surface, further contributing to the hydrophobic behavior [72]. For coatings based on the chitosan/OCL wax composite, the WCA increased with increasing wax content. Previous studies have also reported that lipids are widely used to enhance water repellency in coating systems due to their pronounced hydrophobic characteristics [73]. Furthermore, wax is inherently rich in hydrophobic lipids that resist water penetration, thereby prolonging the water absorption time and improving the overall water resistance of the coated fabrics [74]. The WCA gradually increased as the chitosan/OCL wax ratio reached 70/30, achieving a value of 157.87°. This enhancement may be attributed to the excellent emulsifying ability of chitosan, which promotes the formation of stable lipid latexes through electrostatic interactions between positively charged chitosan molecules and negatively charged lipids or wax [75]. However, samples with excessive wax content (50/50, 30/70, and 10/90) exhibited lower WCA values. This reduction may be attributed to wax agglomeration at higher concentrations, which likely disrupted the uniformity and continuity of the coating layer on the fabric surface. The distribution and concentration of wax on the cotton fabric surface play a crucial role in determining the resulting surface hydrophobicity [76].

### 2.6. Mechanical Properties

The mechanical property results are presented in Table 2. The uncoated cotton fabric exhibited a thickness of 0.24 mm, whereas application of the chitosan/OCL wax composite altered the fabric thickness depending on the chitosan-to-OCL wax weight ratio.

The thickness of the chitosan-coated cotton fabric (100/0 sample) increased to 0.27 mm. These results demonstrate that chitosan treatment increased the fabric thickness, in agreement with previous findings [77]. The thickness of the coating layer can significantly influence the mechanical performance of the textile substrate. The deposition of additional layers on the cotton surface may contribute to enhanced tensile strength. Moreover, coating thickness can affect the swelling behavior of the treated fabric. A thicker coating may function as a barrier to liquid penetration, thereby reducing water uptake, whereas a thinner coating may facilitate liquid ingress and consequently increase water absorption. A further increase in thickness (0.29–0.31 mm) was observed after the incorporation of OCL wax, with the degree of thickening depending on the amount of wax applied. This trend is consistent with previous reports [78], which correlated increased film thickness with higher wax concentrations. The more pronounced thickness observed in wax-treated samples is likely attributable to the accumulation of solidified wax on the fabric surface, in contrast to the relatively thinner film formation or pore-filling effect associated with chitosan treatment.

The tensile stress–strain test is the most commonly used mechanical evaluation for fabrics. It assesses the behavior of a fabric specimen under axial tensile loading, enabling determination of the tensile strength and the elongation at break [79]. The tensile strength and elongation at break of the uncoated and coated cotton fabric samples were measured in both the warp and weft directions, as shown in Table 2. The uncoated fabric exhibited tensile strengths of 12.48 and 9.01 MPa in the warp and weft directions, respectively. After coating with chitosan (100/0 sample), the tensile strength increased to 13.02 MPa (warp) and 9.34 MPa (weft). This improvement can be attributed to interactions between chitosan and cellulose. The reactive amino (-NH_2_) groups of chitosan interact with the hydroxyl (-OH) groups of the cotton matrix, creating strong interfacial bonding. In addition, the chitosan coating fills void spaces within the fabric structure and enhances durability, thereby improving stress transfer throughout the fabric [80]. The 90/10 sample exhibited tensile strength comparable to that of the 100/0 sample. The 70/30 sample showed higher tensile strengths of 14.66 MPa (warp) and 13.22 MPa (weft) than both the 100/0 and 90/10 samples. In contrast, the 10/90 sample displayed tensile strength similar to that of the uncoated cotton fabric. These findings indicate that chitosan plays a key role in enhancing the mechanical properties of the cotton fabric, whereas the wax has little influence because it mainly deposits on the fabric surface rather than reinforcing the fiber structure.

Elongation at break is an important mechanical property that indicates the ability of a fabric to stretch before rupture and is expressed as a percentage of its original length. The uncoated cotton fabric showed elongation at break values of 10.50% and 14.00% in the warp and weft directions, respectively. The coated fabrics exhibited elongation at break in the ranges of 9.50–14.99% (warp) and 13.50–18.00% (weft), depending on the chitosan/OCL wax weight ratio. Overall, the coated samples demonstrated lower elongation in the warp direction than in the weft direction. This behavior suggests increased stiffness of the coated cotton fabrics due to crosslinking within the inter-fiber spaces, which restricts chain slippage under applied stress [56].

### 2.7. Stiffness Properties

Fabric stiffness, defined as a fabric’s resistance to bending, is commonly evaluated through bending length and flexural rigidity. These parameters are critical for assessing material performance across various applications [81]. Accordingly, measuring bending length and flexural rigidity allows manufacturers to enhance product comfort, functionality, and durability [82]. Furthermore, bending length serves as a direct indicator of fabric stiffness, with higher values corresponding to greater stiffness. The bending length and flexural rigidity of uncoated cotton fabric and cotton fabrics coated with a chitosan/OCL wax composite are summarized in Table 3.

According to Table 3, the cotton fabrics demonstrate good flexibility, as indicated by bending lengths of 4.12 cm in the warp direction and 3.48 cm in the weft direction. The corresponding flexural rigidities are 1204.34 mg.cm and 725.76 mg.cm, respectively. This behavior can be attributed to the relatively loose woven structure of the fabric. The bending length and flexural rigidity of the chitosan coated cotton fabric were higher than those of the uncoated fabric, indicating increased stiffness after coating. This effect is attributed to the film-forming ability of chitosan. Moreover, flexural rigidity increased slightly following coating with the chitosan/OCL wax composite. The coating treatments also resulted in progressive increases in fabric stiffness with increasing wax content. This behavior is associated with the formation of a surface film, as both wax and chitosan are high molecular weight materials that deposit on the fabric surface, thereby enhancing its rigidity. This enhancement is attributed to the film forming ability of high molecular weight chitosan [83].

### 2.8. Washing Durability

**Figure 7:**
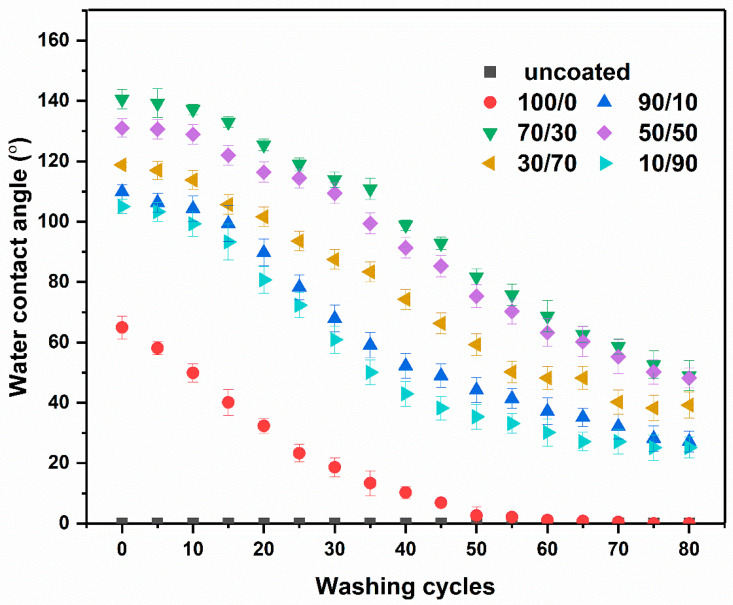
Water contact angles of cotton fabrics coated with a chitosan/OCL wax composite after washing, measured at a holding time of 60 s.

**Figure 8:**
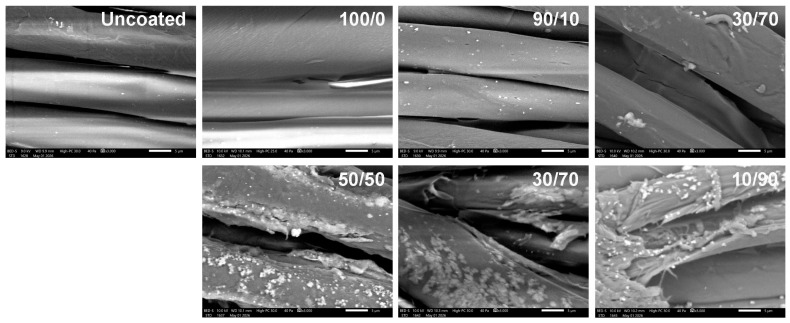
SEM photographs of cotton fabrics coated with a chitosan/OCL wax composite after 80 washing cycles.

Durability is a critical parameter in evaluating the technical performance of water-resistant agents. During laundering, garments are exposed to heat, mechanical forces, detergents, chemical agents, and alkaline conditions (pH 9–11) [84], all of which contribute to the degradation of the water-resistant coating, leading to a gradual decline in hydrophobicity. The effect of washing to the WCA of the cotton fabrics coated with a chitosan/OCL wax composite is shown in Figure 7. SEM images of the coated fabrics with a chitosan/OCL wax composite after 80 washing cycles are present in Figure 8.

As demonstrated in Figure 7, the WCA of the cotton fabrics coated with a chitosan/OCL wax composite gradually decreased with increasing washing cycles. After undergoing 20 washing cycles, the coated fabric still demonstrates hydrophobicity, with a contact angle of 125.44°, For the 30/70 sample. This phenomenon is attributed to strong hydrogen bonding among chitosan, wax, and the fabric. Beyond this point, the WCA progressively declined, reaching 49.00° after 80 cycles. The decrease in WCA can be attributed to the repeated washing, which caused loss of film continuity and partial detachment of the coating from the fabric surface, as shown in Figure 8. Overall, these results indicate that the chitosan/OCL wax composite possesses good resistance to washing.

## 3. Materials and Methods

### 3.1. Materials

Freshly OCL used for wax extraction were collected from Huai Khwang market in Bangkok, Thailand. High-molecular-weight chitosan, with an average molecular weight of 2100 kDa and a deacetylation degree of 90%, was obtained from Marine Bio Resource Co., Ltd. (Samutsakhon, Thailand). Plain weave cotton fabrics were purchased from Dobbytex (Thailand) Co., Ltd. (Bangkok, Thailand) and subsequently scoured in a sodium carbonate solution. Dichloromethane and ethanol were supplied by Chemical Co., Ltd. (Bangkok, Thailand).

### 3.2. Extraction of Wax from OCL

OCL were rinsed thoroughly with tap water, oven-dried at 65 °C for 2 h, and then cut into small pieces (approximately 2 × 2 cm^2^). A 100 g portion of OCL was immersed in 200 mL of dichloromethane in volumetric flask and maintained at 30 °C for 7 days to extract the wax. The resulting extract was filtered, and the solvent was removed using a rotary evaporator (Model R-114, Büchi, Flawil, Switzerland). The obtained wax was decolorized by stirring in ethanol for 1 h, followed by centrifugation. Finally, the decolorized wax was oven-dried overnight at 60 °C.

### 3.3. Preparation of Chitosan/OCL Wax Composite

Six coating composites were prepared with different weight fractions of chitosan to OCL wax (100:0, 90:10, 70:30, 50:50, 30:70, and 10:90). Initially, chitosan was dissolved in a 1% acetic acid solution at 65 °C for 4 h. The OCL wax was then added to the chitosan solution, and the resulting mixture, with a total solid content of 1 wt%, was continuously stirred for 4 h. The homogenously mixed suspensions of chitosan/OCL wax composite are referred to as “X/Y,” where X and Y are the weight ratio of the chitosan and OCL wax in the suspension.

### 3.4. Coating Cotton Fabrics with Chitosan/OCL Wax Composite

Cotton fabrics were coated with a chitosan/OCL wax composite using a dip-coating technique. A coating bath was prepared by mixing chitosan with OCL wax. The fabric samples were immersed in the composite solution and subjected to ultrasonication for 1 h. After coating, the fabrics were oven-dried at 100 °C for 1 h and subsequently stored in a desiccator at 30 ± 3 °C prior to further characterization.

### 3.5. Characterization

#### 3.5.1. Chemical Structure

The functional groups present in chitosan, OCL wax, and cotton fabrics coated with the chitosan/OCL wax composite were examined with FTIR spectroscopy. All spectra were collected on a PerkinElmer Spectrum Two FTIR spectrometer (Shelton, CT, USA). Prior to analysis, the samples were cut into small pieces (~1 mm) and placed directly on the ATR crystal. Spectra were recorded over the range of 4000–600 cm^−1^ with 16 scans at a resolution of 2 cm^−1^.

#### 3.5.2. Colorimetric Analysis

The colorimetric properties $L^{*}$, $a^{*}$, and $b^{*}$ of the uncoated and coated cotton fabric samples were analyzed using a HunterLab UltraScan VIS spectrophotometer (Reston, VA, USA) under a D65 illuminant and a 10° standard observer. Each fabric sample was measured at three different locations, and the average values were reported. The color coordinates L*, a*, and b* were evaluated according to the CIELAB color system, where $L^{*}$ represents lightness on a scale from darkness (0) to lightness (100), $a^{*}$ indicates the red–green axis (+$a^{*}$ = red, −$a^{*}$ = green), and $b^{*}$ represents to the yellow–blue axis (+$b^{*}$ = yellow, −$b^{*}$ = blue).

The total color difference (∆E) between the uncoated and coated fabrics was calculated using Equation (1) [85]:

(1) $$∆E=\sqrt{{\left(L_{0}^{*}{-L}^{*}\right)}^{2}+{\left(a_{0}^{*}{-a}^{*}\right)}^{2}+{\left(b_{0}^{*}{-b}^{*}\right)}^{2}}$$

where $L_{0}^{*}$, $a_{0}^{*}$, and $b_{0}^{*}$ are the color coordinates of the uncoated fabric, and $L^{*}$, $a^{*}$, and $b^{*}$ are those of the coated fabric.

The whiteness index (WI) of the fabric samples was determined using the Hunter whiteness index Equation (2) [86]:

(2) $$whiteness\;index\left(WI\right)=L^{*}-3b^{*}$$

The yellowness index (YI) was calculated according to ASTM E313-1973 [87], using Equation (3) [88]:

(3) $$yellowness\;index\left(YI\right)=\left(100\left(C_{x}X-C_{z}Z\right)\right)/Y$$

where X, Y, and Z denote the measured tristimulus values of the sample, and $C_{x}$, $C_{z}$ are constants determined by the illuminant and observer settings.

#### 3.5.3. Morphological Study

The surface morphology and fracture surfaces of the uncoated cotton fabric and cotton fabric coated with chitosan/OCL wax composite were examined using a JSM-IT210 scanning electron microscope (JEOL, Tokyo, Japan) scanning electron microscope. Fabric samples were securely mounted on a specimen holder with carbon tape to prevent movement during handling. A thin gold coating was applied to improve conductivity and optimize electron–beam interaction. Micrographs were captured at magnifications of 3000× to characterize the surface features.

#### 3.5.4. Air Permeability and Water Vapor Permeability

The air permeability of uncoated and coated cotton fabrics was determined using an air permeability tester (Model M021A, SDL Atlas, Rock Hill, SC, USA) in accordance with ISO 9237:1995 standard [89]. Fabric specimens were cut into 6 cm^2^ samples and conditioned by drying at 60 °C for 24 h prior to testing. Measurements were performed using a circular test head with an effective test area of approximately 5 cm^2^ under an applied air pressure of 100 Pa. Air permeability was expressed as the volume of air passing through the fabric per unit area per second (cm^3^/cm^2^/s).

The water vapor permeability (WVP) was measured using a water permeability tester (Model M261, SDL Atlas, Rock Hill, SC, USA) in accordance with the BS 7209:1990 standard [90]. Fabric samples were prepared as circular samples with a diameter of 10 cm, dried at 60 °C for 24 h, and stored in a desiccator prior to testing. During the measurement, the effective exposed test area had a diameter of 9 cm. The assemblies were mounted on a rotating turntable operating at 2 rpm for a test duration of 16 h. The WVP values, expressed in g/m^2^/day, were calculated using the following equation [91]:

(4) WVP=24 M/At

where M is the weight loss of the test assembly over the measurement period (g), t is the time interval between successive weightings (h), and A is the exposed area of the fabric or the internal area of the test area (m^2^).

#### 3.5.5. Water Contact Angle

The surface wettability of uncoated and coated cotton fabrics was determined using water contact angle measurements (Model OCA 15EC, DataPhysics Instruments GmbH, Filderstadt, Germany). A droplet of water was deposited on the surface of a sample, and the contact angles were recorded over a holding time ranging from 0 to 120 s.

#### 3.5.6. Mechanical Properties

The mechanical properties of uncoated and coated cotton fabrics were evaluated in both the warp and weft directions using a universal testing machine (Instron 5965, Norwood, MA, USA) in accordance with the ASTM D5035-95 standard (strip method) [92]. Rectangular specimens measuring 150 mm × 25 mm were prepared along each fabric direction. Tensile tests were conducted using a gauge length of 75 mm and a crosshead speed of 300 mm min^−1^. Prior to testing, all specimens were conditioned in a desiccator at room temperature (30 ± 3 °C) for 24 h.

#### 3.5.7. Stiffness Properties

The fabric stiffness was measured in accordance with the ASTM D1388-14 standard [93]. Four fabric specimens, each with dimensions of 75 mm × 25 mm, were placed horizontally on the flat surface of a Shirley Stiffness Tester (Shirley Development Ltd., Stockport, England, UK), with one edge aligned with the zero mark on the right-hand side of the instrument. The sliding platform was slowly advanced until the specimen bent and made contact with the knife edge at an angle of 41.5°. The length of the fabric extending beyond the edge was recorded as the bending length. The flexural rigidity was then calculated using the bending length and the fabric mass per unit area according to Equation (5) [94]:

(5) $$G={WC}^{3}$$

where G represents the flexural rigidity (mg.cm), W is the fabric weight per unit area (mg/cm^2^), and C is the bending length (cm).

#### 3.5.8. Washing Durability

The durability of the water-resistant properties of the coated cotton fabrics was evaluated using a Launder-O-Meter (Atlas Material Testing Technology LLC, Mount Prospect, IL, USA) in accordance with AATCC Test Method 61-1994 [95], where one Launder-O-Meter cycle is equivalent to five home laundering cycles. Following the washing process, the water contact angles of the samples were measured using the previously described method.

#### 3.5.9. Statistical Analysis

The colorimetric analysis, air permeability, water vapor permeability, water contact angle, mechanical properties and stiffness properties were performed in five replicates, and data were shown as mean ± standard deviation (SD).

## 4. Conclusions

In this study, cotton fabrics were coated with a chitosan/OCL wax composite using a dip-coating method. The plant-based wax was obtained from discarded outer cabbage leaves. The water-resistant properties of the coated fabrics were systematically evaluated. The results indicated that the weight ratio of chitosan to OCL wax significantly influenced the interaction between the coating components and their adhesion to the fabric substrate. The coating also imparted a more yellowish tone to the fabrics. Among the tested compositions, a 30/70 weight ratio was identified as optimal, providing the highest hydrophobicity with a water contact angle of 157.87°, along with the lowest air permeability and water vapor permeability (10.23 cm^3^ cm^−2^ s^−1^ and 911.63 g m^−2^ day^−1^, respectively). The bending length and flexural rigidity of the coated fabrics increased compared to the uncoated samples, indicating enhanced stiffness after coating. Scanning electron microscopy (SEM) revealed notable changes in the surface morphology after coating, clearly indicating the deposition of chitosan and OCL wax on the surface of the fabric. Furthermore, the results indicate that the chitosan/OCL wax composite exhibits good washing durability. Overall, the results highlight the potential of this chitosan/OCL wax composite as an eco-friendly solution for producing water-resistant textiles, with promising applications in household fabrics, packaging, and wearable clothing.

## Figures and Tables

**Table 1:** Colorimetric data of cotton fabrics coated with chitosan/OCL wax composite.

Samples	$L^{*}$	$a^{*}$	$b^{*}$	WI	YI	∆E
Uncoated	102.67 ± 0.53	1.64 ± 0.03	−14.87 ± 0.06	169.35 ± 1.07	−26.88 ± 0.07	-
100/0	95.56 ± 1.33	1.90 ± 0.03	−12.49 ± 0.10	141.47 ± 3.33	−23.66 ± 0.38	7.50 ± 1.78
90/10	92.57 ± 1.69	2.38 ± 0.11	−5.21 ± 2.42	98.93 ± 5.46	−7.97 ± 2.24	14.00 ± 2.76
70/30	90.90 ± 1.09	2.39 ± 0.12	0.51 ± 1.21	95.31 ± 3.83	−0.24 ± 2.81	19.38 ± 1.02
50/50	87.12 ± 0.45	3.00 ± 0.27	4.97 ± 0.65	87.45 ± 1.74	13.57 ± 1.31	25.24 ± 0.72
30/70	86.43 ± 1.71	3.04 ± 0.90	5.22 ± 0.31	87.26 ± 1.34	13.46 ± 0.62	25.87 ± 4.50
10/90	85.99 ± 1.24	2.38 ± 0.21	5.32 ± 0.14	85.33 ± 1.63	12.86 ± 0.76	26.20 ± 0.80

**Table 2:** Mechanical properties of cotton fabrics coated with chitosan/OCL wax composite.

Samples	Mechanical Properties				
	Thickness (mm)	Tensile Strength (MPa)		Elongation (%)	
		Warp	Weft	Warp	Weft
Uncoated	0.24 ± 0.01	12.48 ± 0.54	9.01 ± 0.46	10.50 ± 0.28	14.00 ± 0.76
100/0	0.27 ± 0.07	13.02 ± 0.82	9.34 ± 0.84	14.99 ± 0.42	16.00 ± 1.23
90/10	0.30 ± 0.03	13.13 ± 1.13	9.95 ± 0.75	11.49 ± 0.35	15.50 ± 1.02
70/30	0.30 ± 0.02	14.66 ± 0.66	13.22 ± 0.68	10.50 ± 0.25	15.00 ± 0.65
50/50	0.31 ± 0.04	14.28 ± 1.00	10.59 ± 0.92	12.00 ± 0.67	18.00 ± 0.44
30/70	0.31 ± 0.01	12.84 ± 0.65	9.30 ± 0.56	9.50 ± 0.22	13.50 ± 0.76
10/90	0.29 ± 0.02	12.52 ± 0.74	9.93 ± 1.01	12.00 ± 0.89	16.00 ± 1.48

**Table 3:** Stiffness properties of cotton fabrics coated with a chitosan/OCL wax composite.

Samples	Stiffness Properties				
	Mass (g/m^2^)	Bending Length (cm)		Flexural Rigidity (mg.cm)	
		Warp	Weft	Warp	Weft
Uncoated	172.21 ± 1.78	4.12 ± 0.36	3.48 ± 0.64	1204.34 ± 11.20	725.76 ± 5.36
100/0	184.27 ± 1.56	4.38 ± 0.16	3.56 ± 1.21	1548.38 ± 12.57	831.39 ± 4.26
90/10	184.59 ± 2.48	4.39 ± 0.53	3.58 ± 0.41	1561.26 ± 20.24	846.95 ± 6.24
70/30	184.61 ± 1.97	4.41 ± 0.31	3.61 ± 0.42	1583.33 ± 15.53	868.51 ± 8.35
50/50	185.01 ± 2.31	4.48 ± 0.54	3.61 ± 1.30	1663.53 ± 16.87	870.40 ± 9.42
30/70	184.75 ± 4.68	4.50 ± 0.68	3.62 ± 1.04	1683.53 ± 21.57	876.42 ± 9.15
10/90	184.36 ± 4.32	4.50 ± 0.97	3.63 ± 1.26	1679.98 ± 24.77	881.83 ± 8.41

## Data Availability

The original contributions presented in this study are included in the article. Further inquiries can be directed to the corresponding author.

## Abbreviations

The following abbreviations are used in this manuscript:

OCL	Outer cabbage leaves
WI	Whiteness index
YI	Yellowness index
WVP	Water vapor permeability
AP	Air permeability
WCA	Water contact angle
FTIR	Fourier transform infrared spectroscopy
SEM	Scanning electron microscopy
